# Changes in body composition of patients undergoing hemodialysis during the coronavirus disease 2019 pandemic: a retrospective longitudinal study

**DOI:** 10.1186/s41100-023-00465-4

**Published:** 2023-02-10

**Authors:** Narumi Fukuzaki, Yuta Suzuki, Juri Uchida, Takuya Nakajima, Shohei Yamamoto, Keigo Imamura, Shun Yoshikoshi, Manae Harada, Ryota Matsuzawa, Kentaro Kamiya, Atsuhiko Matsunaga

**Affiliations:** 1 Department of Rehabilitation Sciences, Graduate School of Medical Sciences, Kitasato University, Sagamihara, Kanagawa Japan; 2 Center for Outcomes Research and Economic Evaluation for Health, National Institute of Public Health, Wakō, Saitama Japan; 3 Department of Epidemiology and Prevention, Center for Clinical Sciences, National Center for Global Health and Medicine, Shinjuku, Tokyo Japan; 4 Tokyo Metropolitan Institute of Gerontology, Itabashi, Tokyo Japan; 5 Department of Rehabilitation, Sagami Circulatory Organ Clinic, Sagamihara, Kanagawa Japan; 6 Department of Physical Therapy, School of Rehabilitation, Hyogo Medical University, Kobe, Hyogo Japan

**Keywords:** COVID-19, Hemodialysis, Body weight, Fat mass, Muscle mass

## Abstract

**Background:**

The spread of coronavirus disease 2019 (COVID-19) has dramatically altered the lifestyles of many people worldwide. Several studies reported that body weight of young adults increased during the COVID-19 pandemic; however, weight loss has been observed in the elderly population. Therefore, trends in body composition due to the COVID-19 pandemic may vary depending on the characteristics of the population. This study aimed to investigate the changes in body mass index (BMI), muscle mass, and fat mass before and during the COVID-19 pandemic among patients undergoing hemodialysis.

**Methods:**

In this retrospective longitudinal study, we enrolled 115 clinically stable outpatients (mean age: 65.7 ± 11.2 years, 62.6% men) who underwent hemodialysis thrice a week. Baseline data were collected between April 2019 and March 2020, before the declaration of the COVID-19 emergency by the Japanese government. The follow-up measurements were performed between July 2020 and March 2021 during the COVID-19 pandemic. Patient characteristics, laboratory data, and BMI measurements were collected from the medical records. Muscle mass and fat mass were measured using bioelectrical impedance analysis.

**Results:**

BMI and fat mass among the study participants were significantly higher during the COVID-19 pandemic than before the pandemic (*p* < 0.01), but no significant change in muscle mass was observed. A restricted cubic spline function showed that the increase in BMI appeared to correlate well with fat mass, but not with muscle mass.

**Conclusions:**

BMI and fat mass of patients on hemodialysis significantly increased due to preventive measures against the COVID-19 pandemic in Japan. These findings may provide useful information in making nutritional management decisions for patients undergoing hemodialysis during and after the COVID-19 pandemic.

## Background

The spread of coronavirus disease 2019 (COVID-19) has dramatically altered the lifestyles of many people worldwide. Lockdown, one of the measures taken to prevent the spread of infection, led to a decrease in physical activity levels due to restrictions in going out. It also resulted in changes in eating habits, which have had a significant impact on weight changes. Generally, body weight has been reported to increase during the COVID-19 pandemic. In contrast, weight loss has been reported in the elderly population [1]. Therefore, the impact of this pandemic is likely to vary depending on the characteristics of the population; this includes weight gain in young adults, and the risks of weight loss, malnutrition, or sarcopenia in the elderly population.

Patients with end-stage renal disease undergoing dialysis are older and at a higher risk of sarcopenia. In particular, weight loss and chronic malnutrition have been shown to worsen the survival of older adults [2]. Therefore, monitoring the impact of the COVID-19 pandemic on body composition is crucial for implementing disease management strategies for this population. However, to the best of our knowledge, no study has identified changes in body composition before and during the COVID-19 pandemic in hemodialysis patients. Therefore, this study aimed to investigate the changes in body weight, muscle mass, and fat mass among patients undergoing hemodialysis, before and during the COVID-19 pandemic.

## Methods

### Patients and study design

This retrospective longitudinal study enrolled clinically stable outpatients who underwent maintenance hemodialysis therapy thrice a week between April 2019 and March 2021 at a Japanese dialysis center. We only included patients who had been assessed both before and during the COVID-19 pandemic so as to focus on the changes in body composition. We used the results of the physical functional assessments conducted in April 2019–March 2020 as pre-pandemic body composition data. In addition, the results of the physical functional assessment conducted in July 2020–March 2021 were referenced as post-pandemic body composition data. Patients were excluded from this study if they had been hospitalized within 3 months prior to the body composition measurement at the baseline; had a cardiac pacemaker; had severe dementia; or had missing data regarding patient characteristics, body mass index (BMI), muscle mass, fat mass, laboratory data, physical function, and physical activity. We identified a total of 226 Japanese hemodialysis outpatients for whom body composition data had been measured before the COVID-19 pandemic. From these, we excluded 111 patients who could not undergo measurement of body composition data at endpoint (during the COVID-19 pandemic). Finally, 115 patients were included in the study.

### Patient characteristics

Patient characteristics including age, sex, dialysis vintage, primary kidney disease, comorbidity, laboratory data, and frailty were collected from the medical records. Comorbidity and frailty were evaluated using the new comorbidity index [3] and Japanese version of the Cardiovascular Health Study (J-CHS) criteria [4], respectively. J-CHS includes five domains: weight loss, exhaustion, physical activity, weakness, and slowness. The index consists of 5 items and they are rated as 0 or 1 by the participants. A score of 3 or more is defined as frail, 1 to 2 as prefrail, and 0 as robust.

Handgrip strength (HGS) was measured using a dynamometer (TKK 5101 Grip-D; Takei, Tokyo, Japan) twice on each side. The highest value (expressed as the absolute value in kg) was used in the analyses. The quadriceps isometric strength (QIS) was evaluated using a handheld dynamometer (μtas F-1; Anima, Tokyo, Japan). Thereafter, the maximum voluntary isometric knee extensor strength was measured three times [5, 6]. Maximum QIS was expressed as a percentage of dry weight (DW), that is, the average of the right and left maximum isometric leg strength divided by DW (% DW). Physical activity level in daily life was measured using an accelerometer (Lifecorder; Suzuken, Nagoya, Japan) [7, 8]. In this study, physical activity was assessed in terms of steps per day. Measurements for four consecutive non-dialysis days were analyzed using the seven-day data.

### Body composition

BMI was calculated by dividing the pre-dialysis dry weight by the square of body height. Muscle mass and fat mass were measured using multi-frequency bioelectrical impedance analysis (InBody S10, InBody Japan, Tokyo, Japan) before initiating hemodialysis treatment [9]. InBody S10 is based on multi-frequency BIA (MF-BIA) that analyzes body composition in 5 segments of the body at 6 different frequencies (1, 5, 50, 250, 500, and 1,000 kHz) [10].

The measurements were performed in a sitting position with the legs set apart and the arms not touching the torso after all metal objects were removed. Patients had electrodes placed on both ankles and the thumbs and middle fingers of both hands to measure muscle mass and fat mass.

### Ethics and consent

This study was performed after obtaining informed consent from all the participants in accordance with the ethical principles of the Declaration of Helsinki and was approved by the institutional research ethics committee. This study was approved by the Research Ethics Committee of Kitasato University (2017-026B-3).

### Statistical analysis

Differences in BMI, muscle mass, and fat mass before and during the COVID-19 pandemic were examined using the Wilcoxon signed-rank test. Subgroup analyses based on sex, age (< 60 years, 60–74 years, ≥ 75 years), and frailty status (robust, pre-frail, frail) were also conducted to assess the differences in the above-mentioned measurements before and during the COVID-19 pandemic using the Wilcoxon signed-rank test. Moreover, we examined the association between changes in BMI and changes in muscle mass or fat mass using the restricted cubic spline function, adjusted for age, sex, and dialysis vintage. In all analyses, a two-tailed *p*-value < 0.05 was considered statistically significant. We used Stata version 15.1 and JMP Pro 15.2.0 software (SAS Institute Inc., Cary, NC) for statistical analyses.

## Results

### Patient characteristics

The characteristics of all participants at baseline level are shown in Table 1. The mean age of the study population was 65.7 ± 11.2 years, 62.6% participants were men, and the median hemodialysis vintage was 6.0 years.

**Table 1:** Patient characteristics at baseline

	Before COVID-19 (*n* = 115)
Age (years)	65.7 ± 11.2
Male (*n*, %)	72 (62.6)
Dialysis vintage (years)	6 [2–15]
New comorbidity index (points)	6 [4–8]
Primary kidney disease (*n*, %)	
Glomerulonephritis (GN) /cystic kidney disease	39 (33.9)
Diabetes	35 (30.4)
Hypertension	11 (9.6)
Unknown	14 (12.2)
Others	16 (13.9)
J-CHS (*n*, %)	
Robust	21 (18.3)
Prefrail	68 (59.1)
Frail	26 (22.6)

Data for continuous variables are presented as mean ± standard deviation or median [interquartile range]; for categorical variables, as number (percentage) *J-CHS* Japanese version of the Cardiovascular Health Study

Changes in laboratory data, physical function, and physical activity data for all subjects before and during the COVID-19 pandemic are shown in Table 2. No change was observed in serum albumin and serum creatinine levels before and during the COVID-19 pandemic. Triglyceride levels significantly increased compared with those before the COVID-19 pandemic (*p* < 0.03). On the other hand, high density lipoprotein (HDL-C), low density lipoprotein (LDL-C), and total cholesterol (TC) levels were significantly decreased (*p* < 0.009, *p* < 0.002, and *p* < 0.008).

**Table 2:** Changes in blood sampling data, physical function, and physical activity data during the COVID-19 pandemic

	Before COVID-19	During COVID-19	*p* value
Serum albumin (g/dL)	3.8 [3.6–3.9]	3.8 [3.6–4.0]	0.39
Serum creatinine (mg/dL)	11.2 [9.5–12.6]	11.1 [9.2–13.2]	0.23
HDL-C (mg/dL)	58 [46–71]	56 [43–67]	0.009
LDL-C (mg/dL)	82 [72–95]	76 [64–86]	0.002
TC (mg/dL)	166.8 [151.4–179.2]	158.6 [140.8–176.8]	0.008
Triglyceride (mg/dL)	104 [76–142]	112 [83–165]	0.03
Muscle strength			
HGS (kg)	21.9 [17.6–-29.5]	23.1 [16.0–-28.5]	0.52
QIS (%DW)	41.6 [33.9–50.1]	38.7 [30.7–48.3]	< 0.001
Physical activity (*n* = 94)			
Number of steps (steps)	4790.3 [2493.9–-6629.9]	3413.8 [1532.4–5692.3]	< 0.001

*HDL-C* high density lipoprotein; *LDL-C* low density lipoprotein; *TC* total cholesterol; *QIS* quadriceps isometric strength; *DW* dry weight; *HGS* Handgrip Strength

No significant difference in HGS was observed before and during the COVID-19 pandemic, whereas QIS was significantly decreased (*p* < 0.001). Number of steps during the COVID-19 pandemic was significantly lower than baseline for the entire subject population (*p* < 0.001).

### Associations between BMI and muscle mass and fat mass

**Fig. 1:**
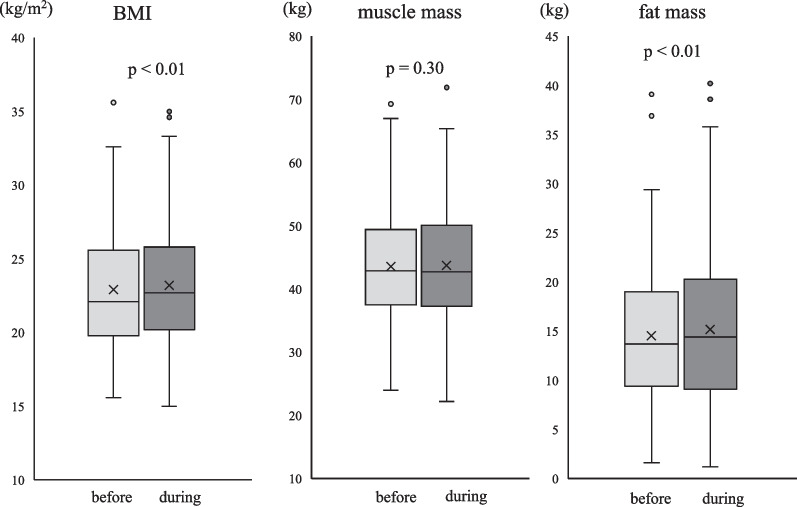
Changes in body mass index (BMI), muscle mass, fat mass before and during the COVID-19 pandemic, and their respective mean values. Muscle mass and fat mass were measured using the bioelectrical impedance analysis method. We used the Wilcoxon signed-rank test to examine changes in BMI, muscle mass, and fat mass. *p*-values indicate the statistical significance of the Wilcoxon signed-rank test

BMI and fat mass during the COVID-19 pandemic significantly increased compared to those before the COVID-19 pandemic in all study participants (*p* < 0.01, respectively) (Fig. 1). In contrast, no significant difference in muscle mass was observed before and during the COVID-19 pandemic (Fig. 1). The results of the subgroup analyses on the changes in BMI, muscle mass, and fat mass before and during the COVID-19 pandemic are shown in Table 3.

**Table 3:** Changes in BMI, muscle mass, and fat mass between before and during the COVID-19 pandemic

	Body mass index (kg/m^2^)			Muscle mass (kg)			Fat mass (kg)		
	Before COVID-19	During COVID-19	*p* value	Before COVID-19	During COVID-19	*p* value	Before COVID-19	During COVID-19	*p* value
Overall (*n* = 115)	22.1 [19.8–25.6]	22.7 [20.2–25.8]	0.01	42.9 [37.5–49.4]	42.7 [37.3–50.1]	0.30	13.7 [9.4–19.0]	14.4 [9.1–20.3]	0.01
Sex									
Male (*n* = 72)	22.9 [20.7–26.4]	23.2 [21.3–27.0]	< 0.001	47.3 [42.9–52.1]	48.1 [43.1–53.8]	0.13	14.4 [8.8–19.7]	16.0 [8.8–20.8]	0.01
Female (*n* = 43)	21.1 [19.0–23.4]	20.8 [19.2–23.5]	0.75	36.5 [32.4–39.1]	36.2 [33.2–39.2]	0.63	11.9 [10.0–17.9]	12.2 [9.1–18.0]	0.75
Age									
< 60 years (*n* = 31)	26.1 [21.8–29.9]	25.4 [22.1–30.1]	0.04	50.2 [41.4–56.9]	50.1 [40.5–55.8]	0.73	17.6 [12.0–25.7]	18.8 [10.4–25.7]	0.07
60–75 years (*n* = 55)	21.4 [19.3–23.8]	21.4 [20.0–23.9]	0.08	42.9 [36.6–48.4]	42.0 [36.2–48.9]	0.51	11.5 [7.8–16.5]	12.2 [8.4–18.9]	0.56
≥ 75 years (*n* = 29)	22.1 [19.2–24.6]	22.6 [19.1–24.5]	0.08	39.7 [34.9–44.4]	40.9 [34.4–43.9]	0.34	14.2 [8.6–18.2]	14.7 [9.2–19.3]	0.12
J-CHS									
Robust (*n* = 21)	21.5 [19.1–25.2]	21.5 [19.3–23.8]	0.30	39.1 [34.3–50.4]	41.2 [34.9–50.2]	0.59	12.4 [10.1–15.7]	12.2 [9.4–17.1]	0.12
Prefrail (*n* = 68)	22.3 [20.6–25.3]	23.0 [20.4–25.3]	0.01	44.1 [37.9–49.7]	43.7 [38.1–50.2]	0.13	14.2 [9.7–19.2]	14.9 [9.4–20.3]	0.14
Frail (*n* = 26)	21.5 [19.0–26.6]	21.3 [19.3–27.1]	0.20	42.0 [37.6–48.9]	40.7 [36.8–48.6]	0.95	13.2 [5.6–21.8]	13.9 [7.8–22.4]	0.33

Data are presented as median [interquartile range]. *p*-values are calculated using Wilcoxon signed-rank test. *J-CHS* Japanese version of the Cardiovascular Health Study

### Associations between change in BMI and muscle mass and fat mass

**Fig. 2:**
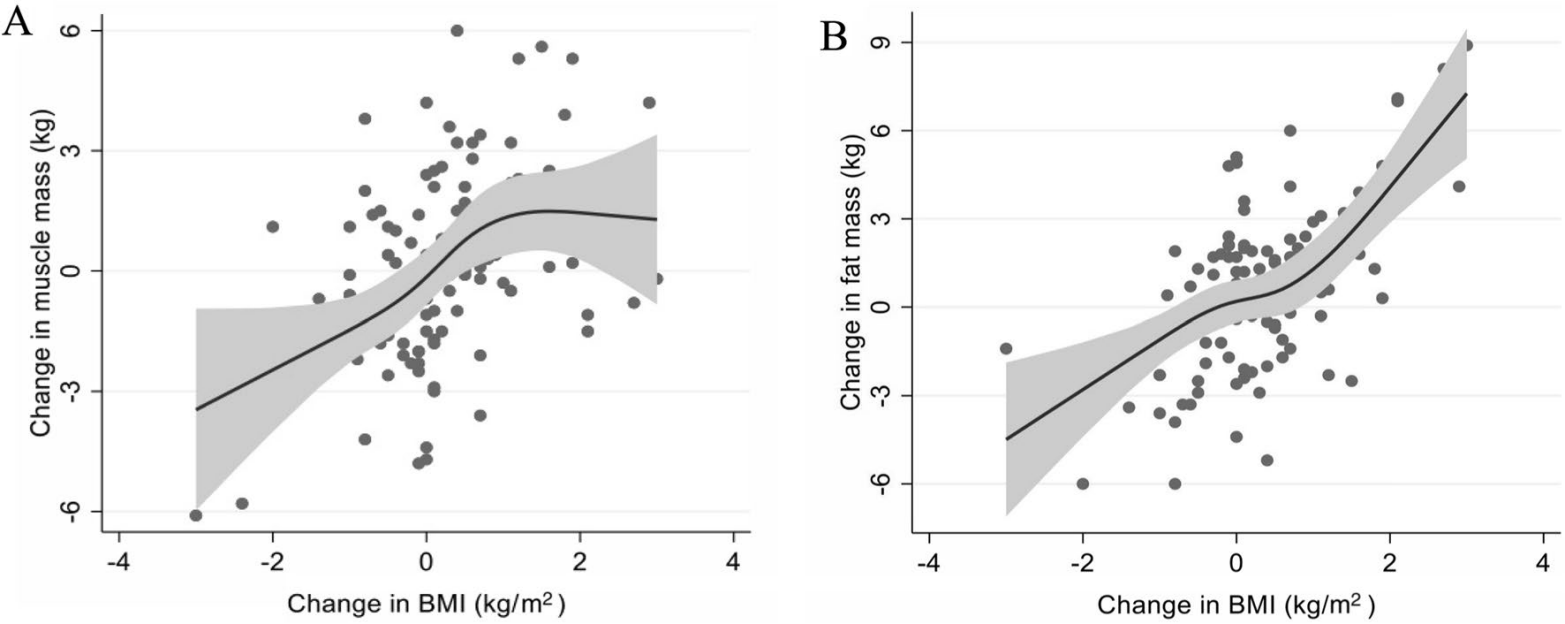
The association between the changes in body mass index (BMI), muscle mass, and fat mass

The correlation between changes in BMI, muscle mass, and fat mass of all participants is shown in Fig. 2A, B. These analyses showed that the increase in BMI (≥ 0%) correlated well with the increase in fat mass, but not muscle mass during the COVID-19 pandemic in patients undergoing hemodialysis.

## Discussion

Our study revealed that BMI and fat mass among hemodialysis patients were significantly higher during the COVID-19 pandemic than before the pandemic, but muscle mass was unchanged. Moreover, our results suggest that the increase in fat mass might result in an increase in BMI because of the association of the fat mass with the BMI, rather than muscle mass.

Prior to the above-mentioned findings, our study showed that physical activity (steps) among hemodialysis patients was significantly lower than before the COVID-19 pandemic. This issue could be considered one of the lifestyle changes that occurred due to the COVID-19 pandemic, suggesting that physical activity of hemodialysis patients in this study was affected by the COVID-19 countermeasures and that it might be not a good environment for hemodialysis patients who have been inactive since before the COVID-19 pandemic. Regarding physical function, although there was no significant change in HGS before and during the COVID-19 pandemic, a significant decrease in lower limb muscle strength after the COVID-19 pandemic was seen in hemodialysis patients.

HGS is an indicator that reflects total muscle mass [11], and this result may support the fact that no change in muscle mass was observed in this study. Although there is no consensus on whether muscle mass or muscle strength is more susceptible to the effects of reduced physical activity, or whether upper or lower extremity muscle mass or strength declines first, it is possible that lower extremity muscle strength was more likely to decline in this study because of the observed decrease in steps per day among hemodialysis patients after the COVID-19 pandemic in this study. A previous study reported that the COVID-19 pandemic restrictions caused muscle mass loss in older patients with type 2 diabetes [12]. Moreover, the reductions in the frequency of going out and physical activity levels due to the COVID-19 pandemic might potentially result in decreases in vitamin D production, insulin sensitivity, and testosterone level, which might lead to a decrease in muscle protein anabolism [13]. Since loss of muscle mass due to decreased physical activity must be prevented for hemodialysis, it is necessary to regularly monitor not only muscle strength but also muscle mass over time.

This study revealed that an increase in BMI was associated with an increase in body fat mass. However, there was no consistent trend in changes in blood lipids before and after the COVID-19 pandemic in this study, indicating that HDL-C, LDL-C, and TC levels decreased, and triglyceride levels increased. Therefore, changes in the blood lipids should be carefully monitored in the future. In addition, we could not determine a causal relationship with increased fat mass because we could not assess changes in the dietary patterns of the study patients. Several studies on body composition during the COVID-19 pandemic noted that the reasons of weight gain were commonly decreased physical activity, decreased energy expenditure, eating more than usual, and increased snacking. Therefore, the changes in the diet pattern may also be closely related to the increase in fat mass in hemodialysis patients.

Although patients undergoing hemodialysis have been shown to lose weight over time due to malnutrition, especially in elderly patients and those with frailty [14], the subgroup analyses in our study showed no significant decreases in BMI in patients aged > 60 years or those with frailty. Moreover, contradictory to our expectations, overall and subgroup analyses showed no change in muscle mass in this study.

Our study provides important insights for nutrition management in patients with hemodialysis after the COVID-19 pandemic. A large longitudinal study stated that body weight and muscle mass of hemodialysis patients have decreased over time [15]. Therefore, we believe that the results in our study reflected outcomes affected by measures in response to the COVID-19 pandemic. Higher BMI is associated with lower mortality risk among patients receiving hemodialysis. Further studies may be needed to examine how the change in BMI during the COVID-19 pandemic affects long-term outcomes in patients on hemodialysis. Study in the U.S. that body weight and muscle mass of hemodialysis patients have decreased over time [15]. Therefore, we believe that our results are similar to the overall phenomena observed during the COVID-19 pandemic. We need to monitor changing trends. Especially, this study observed changes in body composition only for a short period; further studies will be necessary to investigate changes in body composition and to examine whether this trend will have a negative impact on glucose and lipid metabolism in the future, especially since more than half of the patients undergoing hemodialysis have diabetes or cardiovascular disease.

There are several limitations in this study. The first is that body composition assessment using bioelectrical impedance analysis was conducted before dialysis. In addition, the date interval of the measurement during the COVID-19 pandemic was relatively short after the declaration of the state of COVID-19 emergency and we could not conduct sufficient research on socioeconomic factors.

## Conclusions

In conclusion, the significant increases in BMI and fat mass, and no change in the muscle mass of patients on hemodialysis may provide useful information in making nutritional management decisions for patients undergoing hemodialysis. Further continuous studies will be necessary to investigate changes in body composition and to examine whether this trend will have a negative impact in the future.

## Data Availability

Not applicable.

## Abbreviations

COVID-19: Coronavirus disease 2019
BMI: Body mass index
QIS: Quadriceps isometric strength
DW: Dry weight
MF-BIA: Multi-frequency BIA
HDL-C: High density lipoprotein
LDL-C: Low density lipoprotein
TC: Total cholesterol
HGS: Handgrip Strength
